# Infectious disease-associated encephalopathies

**DOI:** 10.1186/s13054-021-03659-6

**Published:** 2021-07-06

**Authors:** Maria C. Barbosa-Silva, Maiara N. Lima, Denise Battaglini, Chiara Robba, Paolo Pelosi, Patricia R. M. Rocco, Tatiana Maron-Gutierrez

**Affiliations:** 1 Laboratory of Immunopharmacology, Oswaldo Cruz Institute, Oswaldo Cruz Foundation, Fiocruz, Av. Brasil, 4365, Pavilhão 108, sala 45, Manguinhos, Rio de Janeiro, RJ 21040-360 Brazil; 2 Anesthesia and Intensive Care, San Martino Policlinico Hospital, IRCCS for Oncology and Neuroscience, Genoa, Italy; 3 Department of Surgical Sciences and Integrated Diagnostics (DISC), University of Genoa, Genoa, Italy; 4 Laboratory of Pulmonary Investigation, Carlos Chagas Filho Institute of Biophysics, Federal University of Rio de Janeiro, Rio de Janeiro, Brazil; 5 National Institute of Science and Technology for Regenerative Medicine, Rio de Janeiro, Rio de Janeiro, Brazil; 6 Rio de Janeiro Network on Neuroinflammation, Carlos Chagas Filho Foundation for Supporting Research in the State of Rio de Janeiro (FAPERJ), Rio de Janeiro, Brazil; 7 National Institute of Science and Technology on Neuroimmunomodulation, Rio de Janeiro, Rio de Janeiro, Brazil

**Keywords:** Sepsis, Malaria, Influenza, COVID-19, SARS-CoV-2, Infection, Neuroinflammation, Microglial priming, Cognition, Encephalopathy

## Abstract

Infectious diseases may affect brain function and cause encephalopathy even when the pathogen does not directly infect the central nervous system, known as infectious disease-associated encephalopathy. The systemic inflammatory process may result in neuroinflammation, with glial cell activation and increased levels of cytokines, reduced neurotrophic factors, blood–brain barrier dysfunction, neurotransmitter metabolism imbalances, and neurotoxicity, and behavioral and cognitive impairments often occur in the late course. Even though infectious disease-associated encephalopathies may cause devastating neurologic and cognitive deficits, the concept of infectious disease-associated encephalopathies is still under-investigated; knowledge of the underlying mechanisms, which may be distinct from those of encephalopathies of non-infectious cause, is still limited. In this review, we focus on the pathophysiology of encephalopathies associated with peripheral (sepsis, malaria, influenza, and COVID-19), emerging therapeutic strategies, and the role of neuroinflammation.

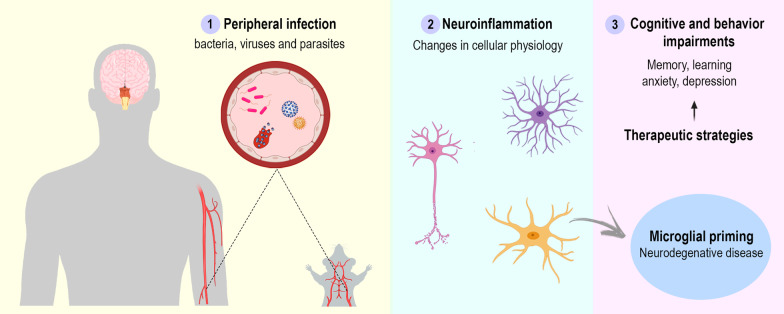

## Background

Encephalopathy is an umbrella term which refers to brain dysfunction, regardless of etiology and pathophysiology. A broad range of diseases are capable of causing encephalopathy, including infections (whether or not the underlying pathogen is able to invade the central nervous system, CNS) (Table 1). Encephalopathies are characterized as temporary or permanent disturbances of brain functions, and the clinical picture is widely variable depending on the etiology [1].

**Table 1:** Major pathogens implicated in infectious disease-associated encephalopathy

Organism	Specific	References
Viruses	Herpes simplex virus	[2]
	Human herpesvirus	[3]
	Influenza A virus	[4]
	Influenza A(H5N1) virus	[4, 5]
	Influenza B virus	[4, 5]
	Human immunodeficiency virus (HIV)	[6]
	Human T-cell lymphotropic virus (HTLV)	[7]
	Chikungunya virus	[8]
	Cytomegalovirus	[9]
	Dengue virus	[10]
	Rift valley fever virus	[11]
	Varicella zoster virus	[3]
	SARS-CoV	[12]
Protozoa	*Toxoplasma gondii*	[13]
	*Trypanosoma cruzi*	[14]
	*Cryptococcus neoformans*	[15]
	*Cryptococcus gattii*	[15]
	*Plasmodium falciparum*	[16]
	*Plasmodium vivax*	[17, 18]
Bacteria	*Klebsiella pneumoniae*	[19]
	*Chlamydia pneumoniae*	[20]
	*Chlamydia psittaci*	[20]
	*Leptospira spp.*	[21]
	*Listeria monocytogenes*	[22]
	*Mycobacterium tuberculosis*	[23]
	*Mycoplasma pneumoniae*	[24]
	*Streptococcus pyogenes* (group A)	[25]
	*Streptococcus* (group B)	[26]

Peripheral infections caused by viruses, bacteria, or parasites may lead to a secondary inflammatory response in the brain, commonly known as neuroinflammation [27], through the action of inflammatory mediators which affect the brain endothelium and parenchyma, and a response of brain cells to these mediators [28]. Therefore, this type of encephalopathy is not considered to be due to direct neurotropism, i.e., invasion of the CNS by the infectious agent. Numerous variables, such as intensity, duration, and immunological imprinting [29], play relevant roles in defining each patient’s outcome; neuroinflammation has been causally linked to long-term neurological damage and to a range of cognitive and behavioral symptoms, including memory loss, cognitive impairment, anxiety, and depression. Indeed, neurological consequences associated with infectious diseases may even influence the future incidence and prognosis of neurodegenerative disorders [30], thus making their proper management a meaningful way of reducing the burden on public health systems. To date, however, there is no established treatment or prevention strategy for the neurological damage associated with peripheral inflammation.

**Fig. 1:**
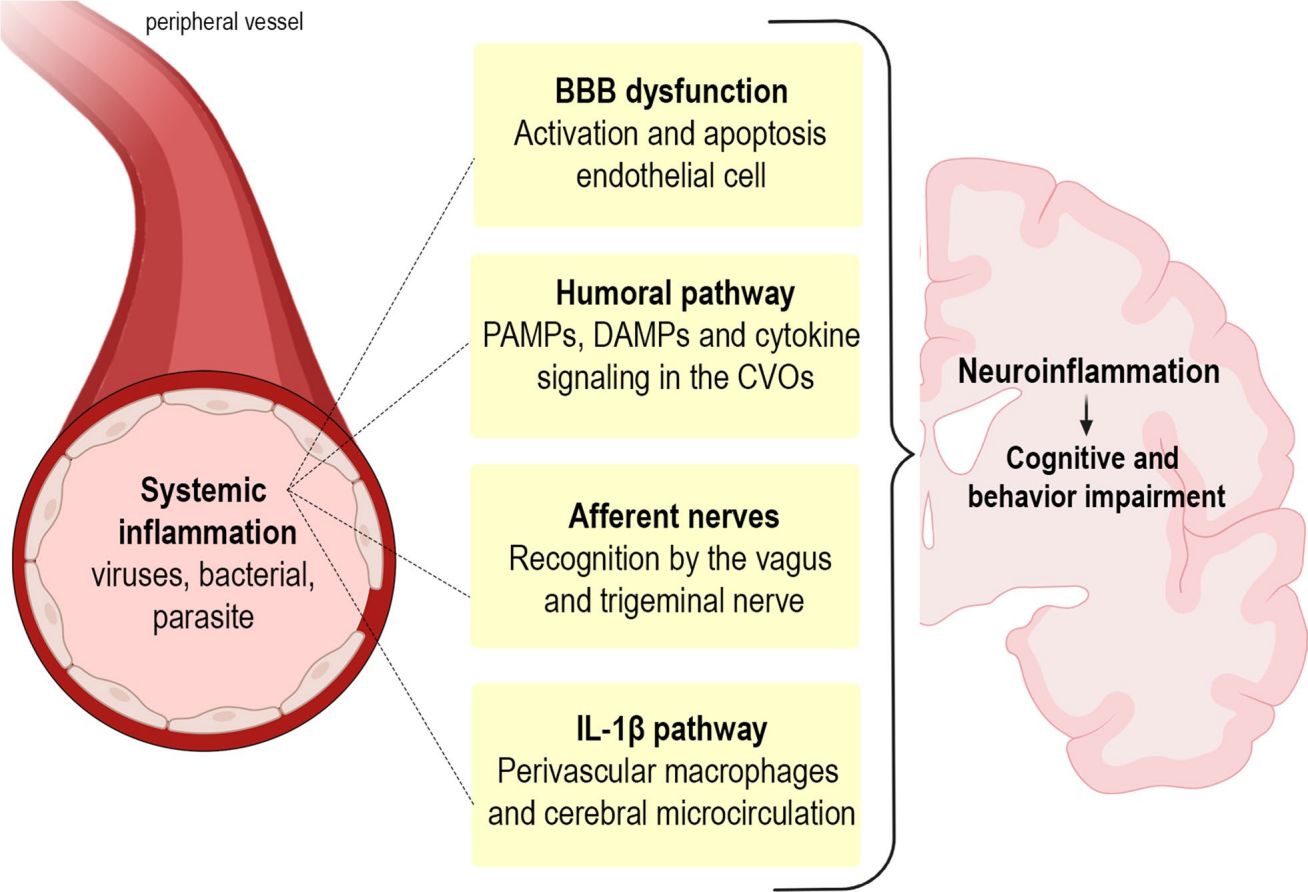
Inflammatory signaling pathways to the brain. Systemic inflammation caused by pathogens, including viruses, bacteria, and parasites, leads to neuroinflammation with consequent cognitive and behavior impairments. The central nervous system is able to recognize systemic inflammation through (1) BBB dysfunction, with activation and apoptosis of endothelial cells, allowing cytokines and immune cells to invade the brain parenchyma; (2) the humoral pathway and saturable transport system in the blood–brain barrier (BBB), which involves the circumventricular organs (CVOs) and the choroid plexus, as local macrophage-like cells express innate immune receptors that recognize pathogen-associated molecular patterns (PAMPs), damage-associated molecular patterns (DAMPs), and cytokines, allowing inflammatory mediators to access the brain by volume diffusion and through cytokine-saturable transporters, since the CVOs do not have an intact BBB; (3) through activation of the afferent nerves (including the vagal nerves in abdominal/visceral infections and the trigeminal nerve in oro-lingual infections) by cytokines; and (4) IL-1β pathway signaling, through activation of IL-1 receptors expressed in perivascular macrophages and endothelial cells located in the brain microvasculature, initiating a local immune response

Peripheral immune responses can crosstalk with the brain through several pathways. Afferent nerves, including the vagal nerves and trigeminal nerves, respond to circulating interleukin (IL)-1β [31–33]. In addition, vagotomized animals do not exhibit sickness behavior after lipopolysaccharide (LPS) or IL-1β injection, despite increased peripheral cytokines levels [34, 35]. The humoral pathway involves macrophage-like cells present in the circumventricular organs and the choroid plexus, which express innate immune receptors that recognize pathogen-associated molecular patterns (PAMP), damage-associated molecular patterns (DAMP), and cytokines. The circumventricular organs do not appear to have an intact blood–brain barrier (BBB); therefore, inflammatory mediators are able to access the brain by volume diffusion, and the cytokine-saturable transporters in the BBB allow the overflowing cytokines present in the peripheral circulation to enter the cerebral parenchyma [31, 36]. The last pathway involves the activation of IL-1 receptors expressed in perivascular macrophages and endothelial cells located in brain microvasculature that initiate a local immune response with local synthesis of prostaglandin E2 [37]. Furthermore, systemic inflammation often leads to an increase in BBB permeability, and, in some cases, frank disruption. The loss of BBB integrity allows cytokines and immune cells to invade the brain parenchyma and directly affect neurons and glial cells [38] (Fig. 1). Glial activation is associated with cognition, memory, and mood disorders, and is a hallmark of neuroinflammation [39, 40].

## Neuroinflammation

Neuroinflammation, an inflammatory condition in the CNS, is a common feature of infectious disease-associated encephalopathies, which is mediated by cytokines, chemokines, reactive oxygen species, among others. These mediators are mainly produced by microglia and astrocytes, endothelial cells, and peripherally derived immune cells. Within the brain, cytokines are able to activate glial cells, modulate neurotransmitter metabolism, and lead to neurotoxic mechanisms [27, 39, 41]. After exposure to pro-inflammatory stimuli, microglia undergo morphological and functional changes, and orchestrate an immune response in the CNS. A pro-inflammatory milieu also leads to several pathological alterations in astroglia. This reactive astrogliosis is characterized by hypertrophy, a modified secretome, and increased expression of intermediate-filament proteins, especially glial fibrillary acidic protein (GFAP) and vimentin [42].

Cytokines exert deleterious effects on the brain, especially the hippocampus. IL-1β inhibits synaptic strength and long-term potentiation in the rodent hippocampus, impacting neuronal morphology, synaptic plasticity [43, 44], and memory and learning processes [45, 46]. Cytokines also affect brain function by modulating neurotrophins. Brain-derived neurotrophic factor (BDNF) signaling is impaired by cytokines, particularly IL-1β [47]. Moreover, systemic injection of LPS has been shown to reduce BDNF, nerve growth factor (NGF), and neurotrophin-3 levels [48], and changes in levels of neurotrophins are known to impact synaptic plasticity, memory, and neuronal survival.

**Fig. 2:**
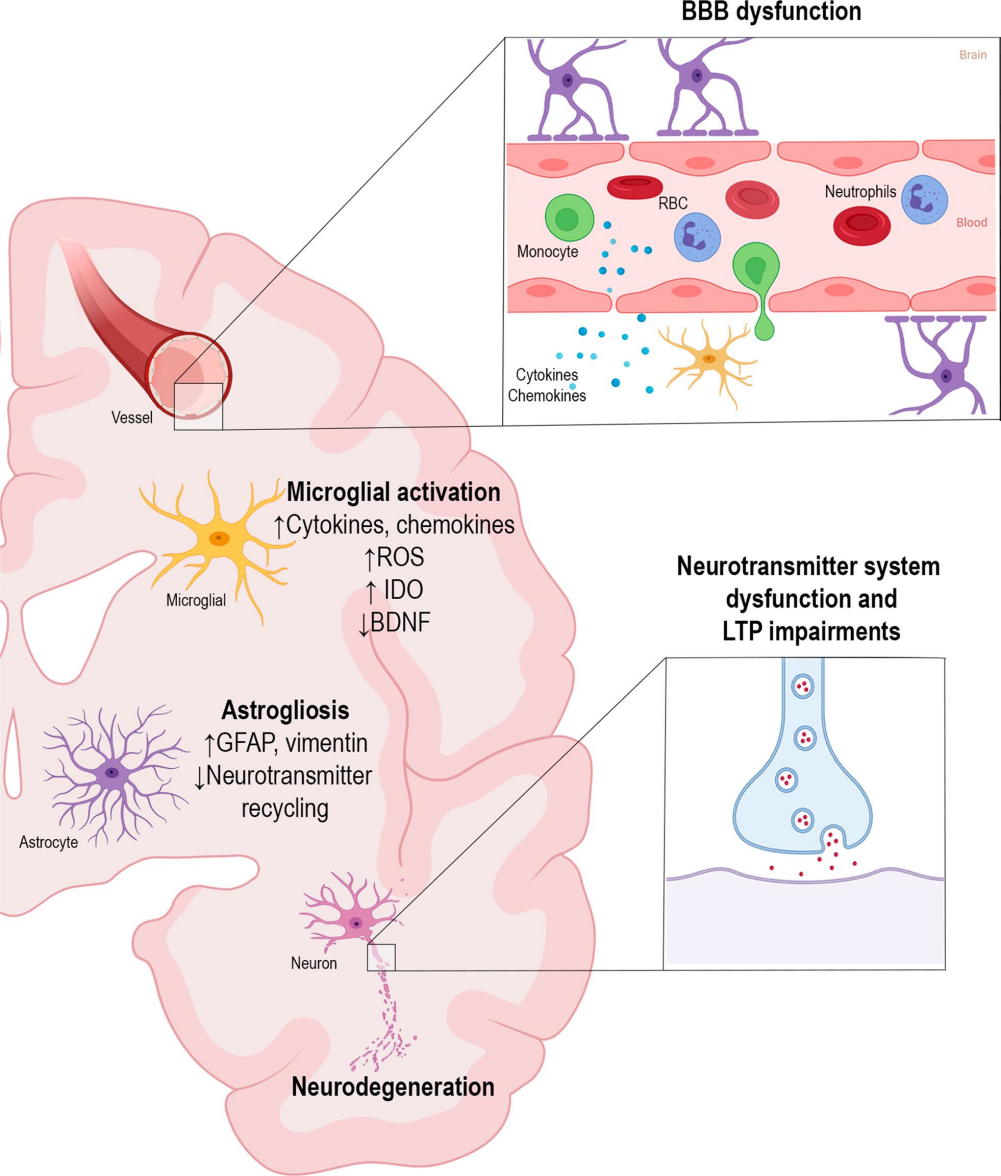
Molecular and cellular mechanisms of neuroinflammation. Blood–brain barrier (BBB) dysfunction contributes to the process of neuroinflammation. After losing its integrity, the BBB allows circulating leukocytes (e.g., monocytes and neutrophils) and proinflammatory mediators, such as cytokines, to enter the brain parenchyma. Microglia and astrocytes proliferate, become reactive, and undergo functional and morphological changes. Microglial cells increase the release of reactive oxygen species, cytokines, chemokines, and indoleamine 2,3-dioxygenase (IDO) expression/activity, as well as decrease brain-derived neurotrophic factor (BDNF) expression. Astrocytes increase the expression of glial fibrillary acidic protein (GFAP) and vimentin, which cause morphological changes, losing their function as supportive glial cells and developing impairment of neurotransmitter recycling. Neuroinflammation also impacts neurons and synaptic transmission, leading to impairments in long-term potentiation (LTP) and neurotransmitter system dysfunctions

Neuronal cells are also affected by glial reactivity and the subsequent loss of the supportive function of glial cells. Astrocytes regulate the concentration of neurotransmitters, such as gamma-aminobutyric acid (GABA), glutamate, and glycine at the synaptic cleft [49]. One of the major consequences of astrogliosis is loss of this function, resulting in glutamate toxicity [39]. Toxicity by glutamatergic activation are also mediated by indoleamine-2,3 dioxygenase (IDO), an enzyme expressed by microglial cells [50]; in the presence of inflammatory mediators, including interferon (IFN)-γ and tumor necrosis factor (TNF)-α, IDO activity is modulated. Moreover, IDO is also involved in tryptophan-serotonin availability suggesting that pro-inflammatory cytokines causes neurotransmitter disbalance [50, 51] (Fig. 2).

## Sepsis-associated encephalopathy

### Definition and diagnosis

The brain is among the multiple organs affected by sepsis [52, 53]. Neurological complications associated with sepsis in the absence of CNS infection fall under the umbrella term sepsis-associated encephalopathy (SAE), which affects 70% of septic patients. It represents a risk factor for mortality, and survivors often face long-term disabilities [54, 55]. In its acute stage, SAE involves sickness behavior, lethargy, delirium, memory impairment, mood disorders, and, in the most severe cases, coma. The diagnosis includes several clinical features such as disturbances in sleep–wake cycles, level of consciousness in disagreement with the dose of sedative received, hallucinations, agitation, and other symptoms of delirium. Moreover, SAE may also lead to paratonic rigidity, and, in 70% of advanced cases, neuromyopathy. Despite these features, SAE is basically a diagnosis of exclusion, with no specific clinical manifestations; it can be inferred and should be suspected after meningitis, encephalitis, and septic emboli from endocarditis have been ruled out. Thus, the final diagnosis relies on the clinical context and evidence of infection in some part of the body [53].

### Pathophysiology and biological alterations

**Fig. 3:**
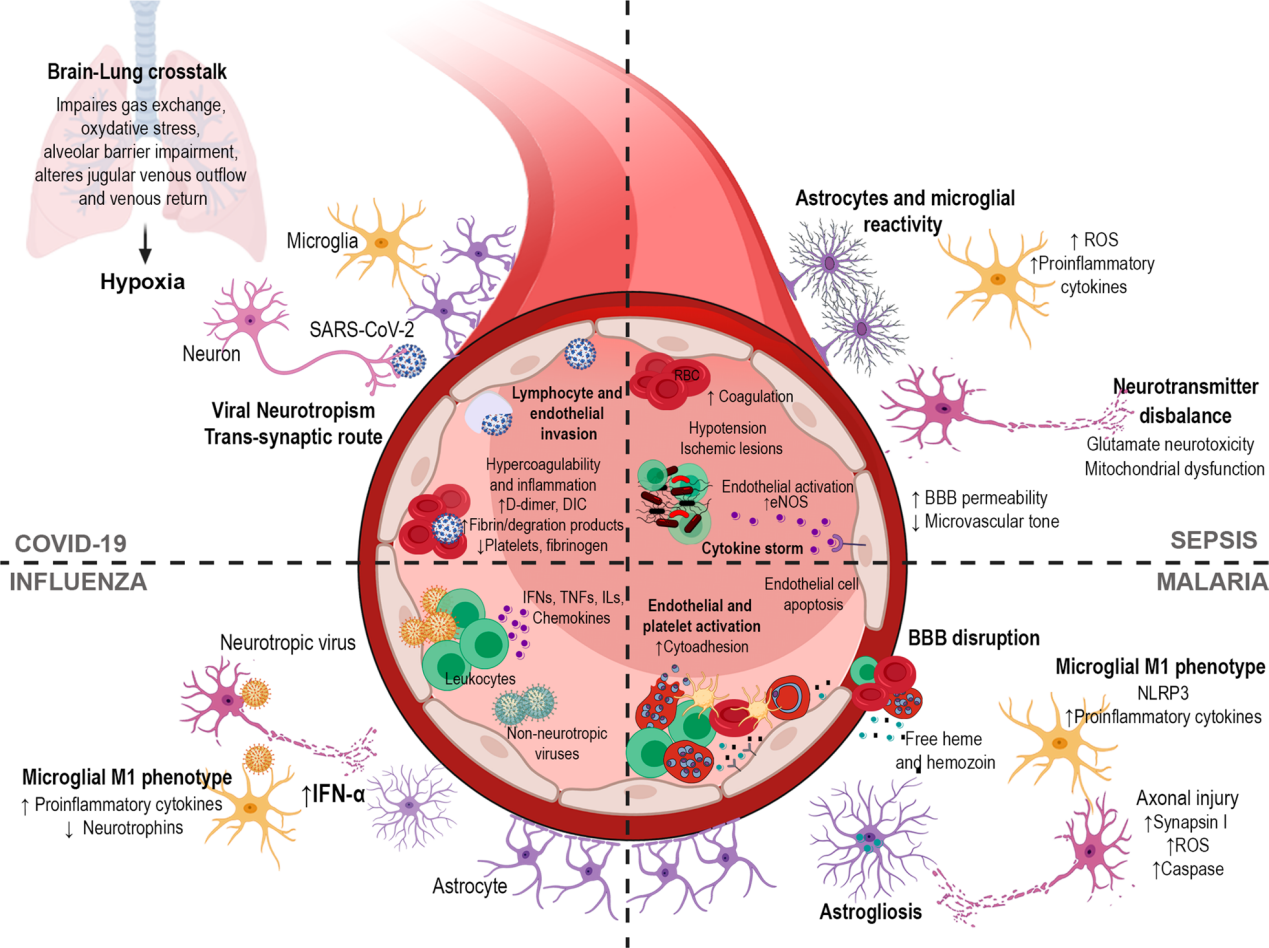
Mechanisms implicated in neurological complications after infection. In COVID-19, SARS-CoV-2 can access the brain by a trans-synaptic route and also through endothelial and lymphocyte invasion, resulting in neuroinflammation. Lower thrombin, higher D-dimer, fibrin/fibrinogen degradation products, and fibrinogen levels are frequent in COVID-19, and activation of the coagulation cascade may contribute to the development of stroke and cerebrovascular accidents. Brain-lung crosstalk is an axis involved in brain hypoxia due to systemic oxygenation reduction and, subsequently, secondary brain oxygenation damage. In sepsis-associated encephalopathy, the cytokine storm leads to endothelial activation and increased eNOS activity, which results in nitric oxide (NO) production, leading to hypotension and ischemic lesions. Cytokines trigger glial reactivity, reactive oxygen species (ROS) production, mitochondrial dysfunction, and neurotransmitter imbalances, with consequent glutamate excitotoxicity. In malaria infection, there is an exacerbated inflammatory response to the parasite and activation of multiple cell death pathways leading to microcirculatory damage. Endothelial dysfunction, platelet activation, cytoadherence, and a downregulation of normal endogenous anticoagulant pathways are hallmarks. Dysregulation of the coagulation pathway leads to microvascular lesions; thrombin may be implicated. In the process of hemoglobin digestion, the malaria parasite releases heme and aggregates it into hemozoin, a highly toxic and proinflammatory signaling molecule. Hemozoin and free heme released into the bloodstream lead to exacerbated inflammation, tissue damage, apoptosis of microvascular brain endothelial cells through activation of STAT3, and loss of BBB integrity through binding to the metalloproteinase MMP3. The proinflammatory milieu leads to microglial M1 phenotype activation, release of proinflammatory cytokines, astrogliosis, axonal injury, and increase in synapsin I. In influenza infection, there is a peripheral inflammatory response and release of several proinflammatory mediators, including interferons (IFs), interleukins (ILs), tumor necrosis factor (TNF), and chemokines. Both neurotropic and non-neurotropic strains of influenza are able to induce neuroinflammation, with microglial activation, decrease in neurotrophin levels, and increase in IFN-α and other proinflammatory cytokines

The pathophysiology of SAE is complex and involves several mechanisms, including neuroinflammation, ischemic processes, neurotransmitter imbalances, and mitochondrial dysfunction [56, 57]. The challenge in defining SAE pathophysiology is the involvement of nonspecific mechanisms and the lack of specific biomarkers. The systemic cytokine storm of sepsis increases BBB permeability and leads to dysfunctions in microcirculation due to exacerbated endothelial-cell activation, resulting in microvascular tone impairment, coagulation activation, and ischemic lesions [58] (Fig. 3). In addition, SAE leads to increased expression and activity of endothelial nitric oxide synthase in neurons and glial cells [59, 60], resulting in augmented NO levels and, consequently, tissue edema and NO-mediated cell death [61, 62].

A decrease in brain volume, especially in the cortex and hippocampus, has been observed in clinical and experimental models of sepsis [63–65]. Damage to these brain areas are associated with impairments in long-term potentiation, affecting learning and memory in models of SAE [66]. Imaging changes can occur in the cortex, subcortical regions, and white matter. Magnetic resonance imaging (MRI) changes are, in the most consistently reported cases, due to cytotoxic edema (caused by hypoxia/ischemia) and vasogenic edema (due to BBB disruption) [67]. In patients diagnosed with some degree of SAE, mortality was directly related to the electroencephalogram (EEG) severity [68].

Changes in neurotransmitter pathways (acetylcholine, GABA, dopamine, norepinephrine, serotonin and glutamate) are considered a hallmark of SAE and are closely related to delirium [69–75]. These changes are in part induced by increased IDO activity [70], but also due to increased plasma levels of the aromatic amino-acid precursors of neurotransmitters, such as tyrosine, tryptophan, and phenylalanine, due to muscular proteolysis and liver failure [71]. These alterations enhance CNS amino acid uptake, which directly impacts neurotransmitter synthesis, leading to abnormalities in neurotransmission [71–73].

During neuroinflammation, several changes occur in cellular metabolism, resulting in mitochondrial dysfunction [76–79], which involve ROS production, increased superoxide dismutase activity [80], energy deficit due to a decrease in adenosine triphosphate (ATP) generation, and cellular apoptosis triggered by the release of cytochrome c [76].

### Therapeutic tools

Sepsis survivors are a complex and heterogeneous group, making it difficult to find a specific therapeutic target. To date, there are still no approaches to prevent or treat the neurological consequences of SAE or the subsequent cognitive decline. In clinical SAE, treatment is primarily symptomatic, despite the fact that these neurological deficits may persist for many years after hospital discharge [77]. Investigation of SAE therapies is a necessary and promising field.

Treatment of delirium requires identification and cessation of any medication with anticholinergic, histaminergic, and other psychotropic properties [78]. Sedatives and neuroleptics must be used with caution, and potent benzodiazepines such as lorazepam must be avoided. In some cases, low doses of neuroleptics may be administered to improve sleep cycles. Dexmedetomidine was associated with shorter duration of clinical encephalopathy, shorter ventilator time, and lower rates of mortality when compared to lorazepam [79]. Considering that the incidence of seizures in SAE is relatively low (10%), antiepileptic drugs should be avoided and only used when justified [81] (Table 2). Immunotherapy with an anti-TNF-α monoclonal antibody reduces mortality in patients with septic shock or high levels of circulating cytokines [82]. Despite this promising finding, there is no evidence that anti-TNF therapy can lead to clinical improvement of SAE.

**Table 2:** Therapeutic approaches to sepsis, malaria, influenza, and COVID-19

Disease	Clinical treatment
Sepsis	Antibiotics for bacterial sepsis: piperacillin/tazobactam, ceftriaxone, cefepime, meropenem, imipenem/cilastatin
	Antiviral drugs for viral sepsis: baloxavir, oseltamivir, peramivir and zanamivir for influenza-associated sepsis; cidofovir for adenoviral infections in immunocompromised patients
	A combination of both antivirals and antibiotics is recommended for viral sepsis
Malaria	Quinine, chloroquine, arthemether-lumefantrine, artesunate, artemisinin
Influenza	Oseltamivir, peramivir, baloxavir, zanamivir
COVID-19	Dexamethasone (mechanically ventilated patients), tocilizumab (non-ventilated patients)

In experimental sepsis, mesenchymal stromal cells (MSC) mitigate BBB dysfunction and neuroinflammation, reduce astrogliosis, and lead to long-term improvements in cognition and anxiety-like behavior [83], as well as resulting in better memory retrieval and decreased sepsis scores at acute time points [84]. Treatment with statins [85], antidepressants [86], and resveratrol [87] reduces microglial activation and prevents long-term cognitive dysfunction [85] and attenuates cognitive and behavioral impairments [86, 87]. Mitochondria is a possible therapeutic target [88] and the use of the mitochondrial division inhibitor Mdivi-1 attenuates oxidative stress and reduces cell death in the hippocampus [89].

## Malaria

### Definition and diagnosis

Malaria is caused by parasites of the gender *Plasmodium* [90]. In 2019, there were an estimated 229 million malaria cases in the world and 409,000 deaths [90]. The proper diagnosis of malaria is essential because identification of the causative *Plasmodium* species is decisive for disease prognosis and choice of therapy. Diagnosis is simple and involves microscopic visualization of parasites in a blood sample or rapid diagnostic tests that detect enzymes or antigens from *Plasmodium*. In countries that have a high prevalence of *P. falciparum*, the causative agent of cerebral malaria (CM), the rapid test for *Plasmodium falciparum* histidine-rich protein 2 (PfHRP2) is commonly used. Severe malaria carries high mortality rates [91, 92] due to complications as metabolic disorders, kidney failure, liver and lung disorders, anemia, and CM [93–96]. Cerebral malaria may lead to neurological complications (seizures, delirium, and coma) as well as cognitive deficits in survivors [97] and is the leading cause of non-traumatic encephalopathy in endemic regions. Non-cerebral malaria may also impact the brain, leading to cognitive and behavioral deficits [17, 98–101].

### Pathophysiology and biological alterations

The pathophysiology of CM involves apoptosis of endothelial cells, BBB rupture, and subsequent neuroinflammation [97], related to an exacerbated systemic inflammation associated with parasite presence and release of toxic molecules, such as heme and hemozoin [101–105]. Additionally, the neurological complications of CM suggest abnormalities in neurotransmitter release. Axonal injury has been observed, thus interrupting neural integrity, distribution of neurosecretory granules, and the transport of enzymes and chemicals involved in the formation of neurotransmitters [103]. Pre-synaptic excitation and activation of synapsin I, a neuronal phosphoprotein that regulates exocytosis of synaptic vesicles and the release of neurotransmitters, have also been reported [104] (Fig. 3).

In patients with CM, brain autopsy shows: (1) cerebral edema, with blood vessels blocked by red blood cells and leukocytes, (2) malarial pigment hemozoin within the vessels, (3) petechial hemorrhages in the white matter, and (4) an abrupt transition from white to gray matter [105]. MRI in CM has revealed: (1) lesions mainly in the frontoparietal lobe, corpus callosum, and internal capsule [106], (2) vasogenic and cytotoxic edema mainly in posterior areas of the brain [107], and (3) focal or diffuse lesions in the centrum semiovale, corpus callosum, thalamus, and cortex [106, 107]. Notably, cases of non-cerebral malaria also show brain changes on MRI [108].

### Therapeutic tools

Although its global impact remains high, malaria is a treatable disease. The main objective of current treatment is to ensure elimination of the parasite. The development of drug-resistant *Plasmodium* strains is a major obstacle to malaria control [109]. Chemoprophylaxis and chemotherapy are currently the only alternatives capable of controlling malaria. Rapid treatment prevents transmission and the progression to severe forms of the disease, including death. The choice of antimalarial drug regimen is largely dependent on the causative species of *Plasmodium*, the severity of the disease, and whether the patient is part of a high-risk group (children, pregnant women, and immunosuppressed individuals) (Table 2). The current first-line treatment for cases of complicated malaria is combination therapy based on intravenous artesunate, artemisinin and its derivatives. Adjuvant therapies such as administration of antipyretics, anticonvulsants, anti-inflammatories, vasodilators, glucose infusion, and blood transfusion are also used in complicated malaria [110]. Routine seizure prophylaxis and induced coma are not recommended in patients with CM. Likewise, the empirical administration of mannitol to reduce intracranial pressure [111] or phenobarbital or fosphenytoin [112] is not recommended. Dexamethasone and other corticosteroids have been shown not to improve vasogenic edema, coma, or recovery, and are therefore not recommended [113, 114].

There are still no therapies to treat the neurological sequelae of CM. Several adjuvant therapies for severe malaria have been tested, such as: rosiglitazone [115–118], statins [119], fasudil, and curcumin [120, 121]. In experimental cerebral malaria (ECM), several therapies have been studied with controversial results [122–139].

## Influenza

### Definition and diagnosis

Influenza is an extremely contagious disease caused by a single-stranded RNA virus and a leading cause of illness and death worldwide, with an estimated of 1 billion cases, and 290,000–650,000 influenza-related respiratory deaths occurring every year [140]. Influenza A and B viruses lead to an acute respiratory infection with fever, cough, chills, myalgia, and headache [141]. Although most patients recover completely from influenza infection, there are short- and long-term consequences in the CNS. The most common extra-respiratory complications are encephalopathies, presenting as delirium, myelopathy, seizures, and ataxia, among other manifestations which usually occur one week after the first symptoms of influenza [4]. Since 1918, various neurological and cognitive effects have been associated with influenza infection. During the 1918 pandemic, several cases of post-influenza psychosis were reported in Europe and the U.S. [122], followed by a nearly decade-long global epidemic of encephalitis lethargica, a complex condition which involves Parkinsonism, lethargy, and sleep disorders [4]. In addition, several cases of other CNS disorders were reported in flu patients, suggesting that influenza may affect the brain and lead to long-term consequences [123]. Influenza-associated encephalopathies and other neurological complications were described in Japan and in several countries following the 2009 pandemic [142]. Fifty percent of patients infected with H1N1 presented neurological symptoms, such as headache, and 9% presented several neurological complications [143]. Moreover, recent outbreaks of seasonal flu have confirmed that neurological complications may arise as a consequence of influenza infections [124]. Nevertheless, the causal link between encephalitis lethargica and influenza remains controversial [125].

The diagnosis of influenza-associated encephalopathy is challenging due to a lack of specific criteria. Detection of influenza RNA in the cerebrospinal fluid, blood samples, and nasopharynx can confirm infection. EEG, brain computed tomography (CT) scan and/or MRI findings, this may suffice to confirm influenza encephalopathy [126, 127]. The major symptoms are headache, numbness, drowsiness, seizures, and, in some cases, coma. Other symptoms such as focal or generalized weakness, vertigo, ataxia, dystonia, and speech disorders have been reported [128, 143].

### Pathophysiology and biological alterations

Some influenza virus strains are considered neurotropic/neurovirulent because they are able to enter the CNS through infection of microvascular endothelial cells or through the olfactory, vagus, trigeminal, and sympathetic nerves. Nevertheless, neurological complications have been reported after infection with neurotropic [129] and non-neurotropic [130, 131] virus strains alike. As most influenza virus strains are considered non-neurotropic, the neurological complications associated with influenza infection likely occur as a consequence of systemic inflammation rather than direct viral invasion [123, 131]. High levels of pro-inflammatory cytokines and chemokines are released into the circulation [144, 145] (Fig. 3). All viral infections, including influenza, elicit a type-I interferon response in the host, which is essential to control the infection [132, 133]. However, increased levels of IFN-α in the brain may contribute to cerebral damage, resulting in memory impairment and depression in humans [134]. In rodents, increased expression of IFN-α leads to neurodegeneration, neuroinflammation, and changes in cognitive function [135]. The non-neurotropic H1N1 influenza strain has been associated with an increase in the hippocampus cytokine levels after infection [130], and spatial memory deficits associated with changes in hippocampal neuron morphology, increased microglial reactivity, and a decrease in neurotrophin expression levels have been reported [131].

In up to 50–55% of individuals with influenza-associated encephalopathy, brain CT scans are normal. MRI may show lesions in the corpus callosum, cerebellum, brain stem, and thalamus bilaterally. Changes in white matter, deep grey matter, and cortical areas may also be seen [127, 136–139].

### Therapeutic tools

There are few studies about therapeutic approaches to treat the neurological complications associated with influenza; in clinical practice, treatment is essentially symptomatic. The main recommendation is to use antiviral treatment as soon as possible to prevent the development of neurological damage [127] (Table 2). The specific mechanism behind this effect remains unclear, but it is presumed that antiviral drugs inhibit viral expression and replication, which results in a diminished inflammatory response [146–148].

There are few reports of a combination of high-dose oseltamivir with glucocorticoids, such as methylprednisolone [149], and dexamethasone [150] with promising results. However, whether oseltamivir reaches sufficient concentrations to inhibit viral replication in the cerebrospinal fluid is unknown [151].

## SARS-CoV-2 infection

### Definition and diagnosis

Severe acute respiratory disease coronavirus 2 (SARS-CoV-2) is a novel coronavirus that has rapidly disseminated worldwide, causing the coronavirus disease 2019 (COVID-19) pandemic [152]. COVID-19 presents a very heterogeneous clinical spectrum from no symptoms to multiple organ dysfunction syndrome (MODS) [12, 153] Neurological symptoms can be present early in the course of the disease [154]; thus, the use of blood biomarkers for diagnosis, such as proteins that have been described to be predictive of brain injury (e.g., S100B), could be helpful [154].

### Pathophysiology and biological alterations

SARS-CoV-2 infects host cells by using its structural proteins—spike (particularly S1), envelope, matrix, and nucleocapsid—to bind angiotensin-converting enzyme-2 (ACE2), a transmembrane protein widely disseminated in the respiratory tract, heart, lung, vessels, kidney, gut, and nervous system. Once bound to ACE2, SARS-CoV-2 is primed by the transmembrane serine protease-2 (TMPRSS2) in two subunits (S1 and S2). The resulting SARS-CoV-2/S1/ACE2 complex is translocated into the target cell, the S2 domain is cleaved, and the genome is released into the cytoplasm. Viral RNA is newly synthetized and replicated, and new viral particles are then assembled and released to infect other cells [155]. Although SARS-CoV-2 enters host cells by endocytosis, three key hypotheses have been proposed for cerebral involvement: (1) direct viral neurotropism; (2) hyperinflammation and hypercoagulation [156]; and (3) brain-lung cross-talk [157, 158] (Fig. 3).

Viral neurotropism may involve binding of SARS-CoV-2 to ACE2 at peripheral nerve terminals, followed by retrograde trans-synaptic passage into the CNS [159]. Other mechanisms include leukocyte migration across the BBB or binding to endothelial cells, allowing the virus to cross the BBB via the microcirculation [157]. Clinically, neurological manifestations of neuro-invasion include smell and taste disorders, which occur in 39.2% of infected individuals [160], as confirmed by MRI findings of cortical hyperintensity in the olfactory bulb and right gyrus rectus [144, 145]. SARS-CoV-2 has also been found in the brain parenchyma at autopsy, as has evidence of a lymphocytic panencephalitis and meningitis [161]. Moreover, infection of the CNS by coronaviruses may be associated with demyelinating, multiple sclerosis-like lesions [162]. However, the majority of cerebrospinal fluid samples are negative for SARS-CoV-2, limiting this hypothesis to few cases of COVID-19-related cerebral involvement [157, 163].

SARS-CoV-2 may pass to the systemic circulation, enhancing the local inflammatory response. Inflammation is a main activator of the coagulation cascade, promoting hypercoagulability, vascular dysfunction, immunothrombosis, and diffuse endotheliitis [157]. The activation of hypercoagulability and pro-inflammation may induce an immune-mediated neuropathology with spontaneous or post-traumatic hemorrhages due to consumption coagulopathy, which can be enhanced by disseminated intravascular coagulation [157]. This hyperinflammatory state can lead to a cytokine storm extending to the nervous system, with possible acute necrotizing encephalitis (ANE) [164]. Stroke can also occur secondary to altered coagulative status in COVID-19 [165–168].

Finally, the brain-lung crosstalk axis is an underestimated mechanism that suggests implications for ventilatory management in the pathogenesis of COVID-19 brain involvement. Reduced systemic oxygenation may affect brain tissue oxygenation, followed by secondary brain damage. Lung derangement may alter the fine balance between oxygen and carbon dioxide [169], an important determinant of cerebral homeostasis because of the changes in cerebral blood flow with consequent brain ischemia or hyperemia [157], eventually causing cerebral edema and loss of cerebral autoregulation [157]. Brain autopsies reported that acute brain hypoxic damage to the cerebrum and cerebellum was present in 100% of COVID-19 deaths, without evidence of encephalitis or brain invasion [170].

### Therapeutic tools

Emerging therapies for COVID-19 include antivirals, immunomodulators, and other agents. No specific therapies have been identified for SARS-CoV-2 brain involvement [12], although general principles regarding neuro-ICU management (such as maintenance of appropriate mean arterial pressure and oxygenation) are warranted. Most of the drugs used against SARS-CoV-2 are currently in clinical trials, and definitive evidence is urgently needed. Direct antiviral activity remains elusive. However, all these drugs do not have a specific effect on the CNS. Dexamethasone has been shown to decrease mortality in patients requiring ventilatory support [171]. The efficacy of corticosteroids in neurological disorders depends on the pathophysiology of the underlying condition. In case of encephalitis or demyelinating lesions, corticosteroids may improve the clinical response, while no recommendation can be made in neurological disorder associated with COVID-19.

The hypercoagulative state characteristic of COVID-19 can be modulated with anticoagulants (e.g., heparin) which have been associated with better prognosis in those with markedly elevated D-dimer [172–175]. In non-ventilated COVID-19 patients, tocilizumab has been included among the medications able to reduce the likelihood of progression to mechanical ventilation or death, but it does not improve survival [176].

Finally, since COVID-19 is characterized by hypoxia, maintaining optimal oxygen delivery by modulating the hemoglobin concentration, the cardiac output, and optimizing ventilator strategies in patients requiring mechanical ventilation may be essential to preventing hypoxic and ischemic brain damage [157, 177] (Table 2).

## Conclusions

To date, no literature review has focused on infectious disease-associated encephalopathies. In this paper, we focused on four infectious diseases known to cause encephalopathy: sepsis, malaria, influenza, and, COVID-19. Observing these infectious diseases caused by different pathogens (bacteria, viruses, and parasites), which present different diagnostic challenges, distinct pathophysiology and different therapeutic approaches allows us to compare the different processes (e.g., cytokine storm, ischemia, alterations in amino acid metabolism) involved in the development of an encephalopathy. Importantly, observing common points shared by these different diseases may help develop new or emerging therapies. Further studies focusing on the treatment of encephalopathies are urgently needed, as therapy remains largely supportive and most experimental studies have yet to reach clinical trials. Lastly, neuroinflammation is a key and common factor between several CNS disorders, including infectious diseases from different etiologies. Thus, the search for therapeutic approaches to address infectious disease-associated encephalopathies must be prioritized to prevent and mitigate additional strain on already overburdened health systems.

## Data Availability

Not applicable.

## Abbreviations

ATP: Adenosine triphosphate
ACE2: Angiotensin-converting enzyme-2
BBB: Blood–brain barrier
BDNF: Brain-derived neurotrophic factor
CLP: Cecal ligation and puncture
CNS: Central nervous system
CM: Cerebral malaria
CT: Computed tomography
DAMPs: Damage-associated molecular patterns
eNOS: Endothelial nitric oxide synthase
ECM: Experimental CM
GABA: Gamma-aminobutyric acid
GFAP: Glial fibrillary acidic protein
HHV: Human herpesviruses
IDO: Indoleamine-2,3 dioxygenase
IFN: Interferon
IL: Interleukin
LPS: Lipopolysaccharide
MRI: Magnetic resonance imaging
MSC: Mesenchymal stromal cells
MPTP: Methyl-4-phenyl-1,2,3,6-tetrahydropyridine
NGF: Nerve growth factor
NO: Nitric oxide
PAMPs: Pathogen-associated molecular patterns
PfHRP-2: *Plasmodium falciparum* Histidine-rich protein 2
pLDH: *Plasmodium* Lactate dehydrogenase
ROS: Reactive oxygen species
SAE: Sepsis-associated encephalopathy
SARS-CoV-2: Severe acute respiratory syndrome-coronavirus-2
STAT: Signal transducers and activators of transcription
TMPRSS2: Transmembrane serine protease-2
TNF: Tumor necrosis factor
